# Association between stress hyperglycemia and acute kidney injury requiring dialysis in critically ill patients with sepsis: a hospital-based cohort study

**DOI:** 10.1590/2175-8239-JBN-2025-0028en

**Published:** 2025-08-08

**Authors:** Marina Borges Wageck Horner, Ed Cleso Pereira de Souza, Gustavo Treichel Schelbauer, Lucas de Oliveira Alves, Helbert do Nascimento Lima

**Affiliations:** 1 Universidade da Região de Joinville, Programa de Pós-graduação em Saúde e Meio Ambiente, Joinville, SC, Brazil.; 2 Universidade da Região de Joinville, Faculdade de Medicina, Joinville, SC, Brazil.

**Keywords:** Renal Failure, Hyperglycemia, Sepsis, Risk Factors.

## Abstract

**Introduction:**

Stress hyperglycemia in patients with sepsis has not been consistently associated with an increased risk of acute kidney injury (AKI).

**Objective:**

To evaluate the effect of blood glucose levels on the occurrence of AKI requiring dialysis in critically ill patients with sepsis.

**Methods:**

Retrospective cohort study of patients with sepsis admitted to the ICU of a private hospital between December 2017 and August 2021. Clinical, laboratory, and severity variables were collected. Mean blood glucose levels in the first week of ICU stay (primary exposure variable) were stratified into tertiles. The effect of blood glucose on the occurrence of dialysis-requiring AKI was assessed using multivariate logistic regression.

**Results:**

Of the 1,317 patients evaluated, 86.6% had clinical conditions as the underlying cause of sepsis. AKI requiring hemodialysis occurred in 12.2% of the sample. Patients with mean blood glucose levels above the third tertile (≥160 mg/dl), compared to those with mean blood glucose levels below the first two tertiles (<160 mg/dl), had a higher prevalence of diabetes (69.1% vs. 7.1%; p < 0.001). Patients with mean blood glucose levels ≥160 mg/dl had a 62% higher odds of developing AKI requiring dialysis compared to those with mean blood glucose levels < 160 mg/dl (crude OR = 1.62; 95% CI 1.16–2.26; p = 0.005). After adjustment for other variables, mean blood glucose levels ≥180 mg/dl did not increase the likelihood of AKI (OR = 1.27; 95% CI 0.76–2.12; p = 0.359).

**Conclusion:**

In this patient group, sepsis and mean blood glucose levels ≥160 mg/dl were not independently associated with the occurrence of dialysis-requiring AKI.

## INTRODUCTION

It is estimated that 30% of intensive care unit (ICU) admissions in Brazil are due to sepsis^1^. Among the complications related to sepsis, stress hyperglycemia and acute kidney injury (AKI) are frequently observed in these patients^2,3^. However, the effect of elevated blood glucose levels associated with stress has not yet been fully evaluated in terms of its independent risk for AKI.

Sepsis is a major cause of ICU admission among adult patients worldwide^1,2,3,4^. A study conducted in 730 ICUs across more than 80 countries showed that the incidence of sepsis during ICU stay was 30%, with 18% already presenting with sepsis at admission^4^. Dysregulated glycemic control, known as stress hyperglycemia or critical illness-related hyperglycemia, is present in 40 to 60% of ICU patients with sepsis^3^ and is associated with an increased 30-day mortality risk, regardless of pre-existing diabetes^5^.

In addition to glycemic dysregulation, sepsis is associated with 40% to 70% of AKI cases in patients admitted to ICUs^2^, and represents a major cause of mortality and increased hospital length of stay^2,3,4,5,6,7^. Van de Berghe et al.^8^ highlighted the importance of a more restrictive treatment of hyperglycemia (80–110 mg/dl), compared to conventional glycemic control (180–200 mg/dl) in critically ill patients in a surgical ICU, in reducing mortality rates, mechanical ventilation time, and ICU length of stay, as well as the occurrence of dialysis-requiring AKI. The same study was replicated in a medical ICU and, despite the lower incidence of AKI in that setting, stricter glycemic control was not associated with a reduced need for dialysis^9^. Experimental studies in animal models have demonstrated the deleterious effect of acutely elevated blood glucose levels on the architecture and homeostasis of renal tubular cells, as well as on the activation of various inflammatory mediators^10^. Studies involving critically ill patients hospitalized for heart failure or myocardial infarction have identified an association between elevated stress-related blood glucose levels and the occurrence of AKI^11,12^. However, this association between acute hyperglycemia and increased risk of AKI, especially in the context of sepsis, has not been consistently observed in studies involving ICU patients^9,10,11,12,13^.

Thus, considering that AKI is a common complication among ICU patients, particularly those admitted for sepsis, the present study aimed to evaluate the association between acute hyperglycemia and the occurrence of AKI requiring dialysis in critically ill patients with well-defined sepsis criteria.

## METHODS

### Design, Location, and Sampling

This was a retrospective cohort study using data obtained from the electronic medical records of patients admitted to the ICUs of the *Centro Hospitalar Unimed* (CHU) in Joinville, SC, Brazil, between December 2017 and August 2021. The CHU is a private general hospital that serves beneficiaries of the Unimed supplementary health system in the region. The hospital has 152 general ward beds and 19 general adult ICU beds. In addition, the CHU offers a medical residency program in intensive care medicine, and all physicians working in the ICU hold a board certification in this specialty. All patients aged 18 years or older who were admitted to one of the CHU’s ICUs with a diagnosis of sepsis and/or septic shock, based on the SEPSE-3 criteria, were included^14^. Patients undergoing palliative care, patients with chronic kidney disease (previous history of chronic renal failure already on dialysis or those with creatinine ≥ 3 mg/dl at hospital admission), patients with dialysis-requiring AKI prior to ICU admission, and those with mild AKI (stage 1) after ICU admission were excluded from the study. Patients with stage 1 AKI were excluded in order to focus exclusively on those with AKI requiring dialysis, since there were no patients with stage 2 or 3 AKI without dialysis requirements in the study sample. This study was approved by the Univille Ethics Committee, under opinion number CAAE 51034021.2.0000.5366. The Free and Informed Consent Form (FICF) was waived, considering the retrospective and observational nature of the study.

### Collected Variables

The variables considered were age, sex, previous diagnosis of diabetes mellitus (DM) as reported by the patient, and reason for ICU admission (clinical or surgical causes). Additionally, the following were included: need for acute hemodialysis, the Simplified Acute Physiology Score 3 (SAPS 3) at ICU admission, mechanical ventilation time and ICU length of stay, as well as use of vasopressor drugs, corticosteroids, and nephrotoxic antibiotics (vancomycin, amphotericin, aminoglycosides, and/or polymyxin B). Insulin use was also considered, following the institutional hyperglycemia protocol, in which two episodes of hyperglycemia > 180 mg/dl would indicate the initiation of intravenous insulin therapy.

### Main Exposure and Outcome Variables

Capillary blood glucose was measured through hemoglucotest (HGT) using a blood glucose meter with test strips, both manufactured by G-Tech®. For patients receiving intravenous insulin, HGT was performed hourly, whereas for the remaining patients, measurements were performed routinely (four times a day). Mean blood glucose levels during the first week of ICU stay were considered the primary exposure variable of the study. The outcome variable assessed was the occurrence of stage 2 or 3 AKI, with or without the need for urgent dialysis. The definition of AKI was based on KDIGO criteria, considering the increase in creatinine relative to baseline values upon admission to the ICU. The indication for dialysis was determined by a nephrologist according to clinical parameters.

### Statistical Analysis

Categorical variables are presented as absolute frequencies and percentages. Numerical variables are expressed as mean and standard deviation, or median and interquartile range. Mean hyperglycemia values in the first week of ICU stay were stratified by tertiles. After verifying non-normal distribution by the Kolmogorov-Smirnov test, quantitative variables were compared using the Mann-Whitney U test and Kruskal-Wallis test, while categorical variables were compared using the chi-square test, stratified by the main exposure of interest and outcome. Subsequently, a bivariate analysis was performed to compare both the crude and adjusted effects on patients with mean blood glucose levels above the third tertile (≥160 mg/dl) in the first week, compared to those with values between the first and second tertiles ( < 160 mg/dl) for the occurrence of dialysis-requiring AKI. This analysis was performed using logistic regression, considering potentially confounding variables. All variables that modified the main effect studied by 5% or more, as well as all those recognized as associated with the main exposure or considered to be risk factors for the outcome, were included in the final multivariate model by logistic regression. A p-value < 0.05 was considered statistically significant. The analyses were performed using STATA/IC statistical software, version 15.

## RESULTS

**Figure 1:**
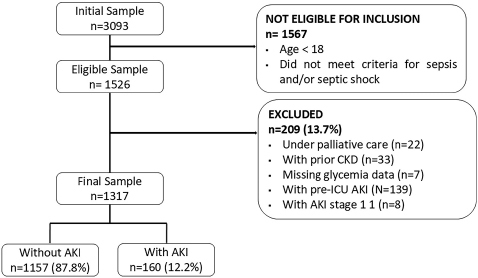
Sampling flow chart.

Of the 3,093 patients admitted to the ICU during the study period, 1,317 met the inclusion criteria and were considered in the analysis, as shown in Figure 1. Of these, 57.6% were aged 60 years or older, and 53.3% were male. Stage 3 AKI was identified in 12.2% of patients, all of whom required urgent hemodialysis. The main reason for ICU admission (86.6%) was related to clinical conditions. When stratified by the presence or absence of AKI requiring dialysis, it was observed that, compared to patients without dialysis-requiring AKI, those requiring dialysis had a higher frequency of age over 44 years (88.1% vs. 79.7%; p = 0.021) and a higher prevalence of males (66.9% vs. 51.4%; p < 0.001). In addition, they had higher median values of C-reactive protein (237 mg/dl vs. 183 mg/dL; p < 0.001), creatinine at hospital admission (1.0 mg/dL vs. 0.8 mg/dL; p < 0.001) and at ICU admission (1.0 mg/dL vs. 0.7 mg/dL; p < 0.001). Patients with dialysis-requiring AKI also needed greater use of vasoactive drugs and corticosteroids, and had longer median times on mechanical ventilation and hospital stay. The remaining characteristics are shown in Table 1.

**Table 1:** General characteristics of the sample and stratified by the presence or absence of acute kidney injury (stage 3)

	Total Sample n = 1317		No AKI n = 1157 (87,8%)		With AKI n = 160 (12,2%)		*p*-Value
	n° total or median	% or VIQ	n° total or median	% or VIQ	n° total or median	% or VIQ	
Age, years							0,021
18–44	254	19.3	235	20.3	19	11.9	
45–59	305	23.2	259	22.4	46	28.7	
≥60	758	57.6	663	57.3	95	59.4	
Sex, male	702	53.3	595	51.4	107	66.9	< 0.001
Comorbidities, yes							
Diabetes	424	32.2	363	31.3	61	38.1	0.087
Covid	56	4.2	47	4.0	9	5.6	0.358
Reason for Admission							0.136
Clinical	1140	86.6	989	86.4	145	90.6	
Surgical	177	13.4	156	13.6	15	9.4	
Tertile of the average blood glucose of the 1st week							0.015
70–127	436	33.1	395	34.1	41	25.6	
128–159	433	32.9	384	33.2	49	30.6	
≥160	448	34.0	378	32.6	70	43.7	
PCR ICU admission, mg/dl	188	66/280	183	62/276	237	83/307	< 0.001
Creatinine hospital admission, mg/dl	0.8	0.6/1.2	0.8	0.6/1.1	1.0	0.7/2.1	< 0.001
Creatinine ICU admission, mg/dl	0.8	0.6/1.1	0.7	0.5/1.1	1.0	0.7/2.1	< 0.001
AKI (stage 3), yes	160	12.1					
HD urgency, yes	160	12.1					
SAPS-3	50	42/60	49	42/59	56	49/66	< 0.001
MV, days	6	1/18	4	1/13	20	11/40	< 0.001
Days in the ICU	8	3/19	7	3/16	25	14/45	< 0.001
Days in the hospital	16	8/30	14	8/28	33	19/57	< 0.001
VAD, yes	586	44.5	484	41.8	102	63.7	< 0.001
Corticosteroid, yes	698	53.0	570	49.3	128	80.0	< 0.001
Nephrotoxic ATB, yes	193	14.6	118	10.2	75	46.9	< 0.001
IV Insulin yes	827	62.8	703	60.8	124	77.5	< 0.001

Abbeviations – VIQ: interquartile variation (25th percentile/75th percentile); CRP: C-reactive protein; AKI: acute kidney injury (stage 2 or 3); SAPS-3: simplified acute physiology score; MV: mechanical ventilation; VAD: vasoactive drugs; ATB: antibiotic; VS: intravenous; TPN: total parenteral nutrition.

Table 2 presents the characteristics of the sample, stratified by tertiles of mean blood glucose levels in the first week of ICU stay. There was variation in age distribution between the mean blood glucose tertiles during the first week. Compared to patients in the first tertile, those in the third tertile had a higher prevalence of age over 60 years (70.8% vs. 43.1%; p < 0.001), previous history of diabetes (69.1% vs. 7.1%; p < 0.001) and a lower prevalence of hospitalization for surgical reasons (8.3% vs. 18.5%; p < 0.001). Furthermore, patients in the last tertile presented higher median creatinine values at hospital admission and in the ICU, higher SAPS-3 severity scores, longer hospital length of stay, and ICU stay, longer mechanical ventilation time, as well as greater need for vasoactive drugs, corticosteroids, and IV insulin. The presence of dialysis-requiring AKI was also higher in the group with higher mean blood glucose levels during the first week, compared to the first tertile (15.6% vs. 9.4%; p = 0.015).

**Table 2:** Sample characteristics stratified by mean tertile of blood glucose in the first week of the ICU

	Tertile 1 (70–127mg/dL) N = 436 (33,1%)		Tertile 2 (128–159mg/dL) N = 433 (32,9%)		Tertile 3 (≥160mg/dL) N = 448 (34,0%)		*p*-Value
	Total or median	% or VIQ	Total or median	% or VIQ	Total or median	% or VIQ	
Age, years							< 0.001
18–44	145	33.3	74	17.1	35	7.8	
45–59	103	23.6	106	24.5	96	21.4	
≥60	188	43.1	253	58.4	317	70.8	
Sex, male	226	51.8	226	52.2	250	55.8	0.424
Comorbidities, yes							
Diabetes	31	7.1	83	19.2	310	69.1	< 0.001
Covid	14	3.2	22	5.1	20	4.5	0.379
Reason for Admission							< 0.001
Clinical	353	81.5	375	87.4	406	91.6	
Surgical	80	18.5	54	12.6	37	8.3	
PCR ICU admission, mg/dl	196	68/286	192	70/290	180	59/269	0.127
Creatinine hospital admission, mg/dl	0.7	0.5/1.0	0.8	0.6/1.1	0.9	0.7/1.4	< 0.001
Creatinine ICU admission, mg/dl	0.7	0.5/0.9	0.8	0.6/1.1	0.9	0.6/1.4	< 0.001
AKI, yes	41	9.4	49	11.3	70	15.6	0.015
SAPS-3	47	39/55	50	42/60	54	46/64	< 0.001
MV, days	2	1/11	7	1/21	9	3/19	< 0.001
Days in the ICU	5	3/14	10	4/21	10	5/20	< 0.001
Days in the hospital	13	7/25	18	9/36	18	9/31	< 0.001
VAD, yes	152	34.9	196	45.3	238	53.1	< 0.001
Corticosteroid, yes	175	40.1	251	58.0	272	60.7	< 0.001
Nephrotoxic ATB, yes	61	14.0	66	15.2	66	14.7	0.871
IV Insulin yes	115	26.4	278	64.2	448	96.9	< 0.001

Abbeviations – VIQ: interquartile variation (25th percentile/75th percentile); CRP: C-reactive protein; AKI: acute kidney injury (stage 2 or 3); SAPS-3: simplified acute physiology score; MV: mechanical ventilation; VAD: vasoactive drugs; ATB: antibiotic; VS: intravenous; TPN: total parenteral nutrition.

When evaluating the effect of higher mean blood glucose levels in the first week (third tertile, ≥160 mg/dl) compared to the first two tertiles (< 160 mg/dl) on the occurrence of dialysis-requiring AKI (Table 3), it was observed that patients within this setting had a 62% higher odds of developing AKI (OR = 1.62; 95% CI 1.16–2.26; p = 0.005). After adjusting this effect on the occurrence of AKI for the other variables, in a bivariate analysis, it was found that mechanical ventilation time, the use of vasoactive drugs, corticosteroids, and nephrotoxic drugs modified this effect by 5% or more. When adjusted for SAPS-3 and need for intravenous insulin, although also with a change in effect of 5% or more, there was a loss of association between higher mean blood glucose values in the first week above the third tertile – compared to the first two tertiles – and the occurrence of AKI requiring dialysis.

**Table 3:** Crude and adjusted odds ratio (bivariate analysis) of the effect of hyperglycemia means ≥160mg/dl on the occurrence of dialysis AKI by logistic regression

	Crude OR	CI 95%	*p-*Value
Effect of mean blood glucose of the 1st week in the first/second tertile vs. third tertile (<160mg vs. ≥160 mg)			
<160 mg/dl	1.00	Reference	
≥160 mg/dl	1.62	1.16-2.26	0.005
Effect of the 1st week glycemic mean on the first/second tertile vs. third tertile (<160mg vs. ≥160mg) adjusted for:	Adjusted OR		
Age, Years; per unit of increase	1.58	1.12–2.22	0.010
Sex, men vs. women	1.59	1.13–2.23	0.007
Diabetes, yes vs. no	1.68	1.19–2.37	0.003
COVID-19, yes vs. no	1.62	1.15–2.26	0.005
CRP ICU admission, mg/dl;per unit of increase	1.64	1.16–2.32	0.005
SAPS-3; per unit of increase	1.38	0.95–1.99	0.090
MV, days; per unit of increase	1.51	1.04-2.21	0.032
VAD, yes	1.46	1.04–2.05	0.030
Corticosteroid, yes	1.43	1.01–2.01	0.042
Nephrotoxic antibiotics, yes	1.72	1.20–2.46	0.003
Intravenous Insulin yes	1.17	0.80–1.70	0.411

Abbeviations – ICU: intensive care unit; CRP: C-reactive protein; MV: mechanical ventilation; VAD: vasoative drugs.

Table 4 shows the final effect of the presence of mean glycemic levels above the third tertile on the occurrence of dialysis-requiring AKI. All variables previously assessed in the bivariate analysis were included in the model, as they were considered potential confounders for the primary association studied. After adjustment for the other variables, the presence of mean blood glucose values in the first week above the third tertile (≥160 mg/dl) did not increase the likelihood of dialysis-requiring AKI (OR = 1.27; 95% CI 0.76–2.12; p = 0.359).

**Table 4:** Multivariate analysis of the effect of mean hyperglycemia ≥160mg/dl on the occurrence of acute kidney injury requiring dialysis by logistic regression

	Crude OR	CI 95%	*p-*Value
Effect of mean blood glucose of the 1st week in the first/second tertile vs. third tertile ( < 160mg vs. ≥160mg)			
< 160 mg/dl	1.00	Reference	
≥160 mg/dl	1 27	0.76–2.12	0.359
Age, Years; per unit of increase	1.00	0.99–1.02	0.618
Sex, men vs. women	1.37	0.86–2.16	0.182
Diabetes, yes vs. no	1.43	0.59–3.52	0.426
COVID-19, yes vs. no	0.98	0.42–2.27	0.963
CRP ICU admission, mg/dl;per unit of increase	1.00	1.00–1.01	0.001
SAPS-3; per unit of increase	1.03	1.01–1.05	0.001
MV, days; per unit of increase	1.02	1.01–1.03	< 0.001
VAD, yes	1.30	0.81–2.08	0.278
Corticosteroid, yes	1.87	1.11–3.16	0.019
Nephrotoxic ATB, yes	4.06	2.47–6.68	< 0.001
IV Insulin yes	1.17	0.65–2.10	0.589

Abbeviations – ICU: intensive care unit; CRP: C-reactive protein; MV: mechanical ventilation; VAD: vasoative drugs.

## DISCUSSION

The present study, based on a retrospective cohort of critically ill patients with well-defined sepsis criteria, found no association between mean blood glucose levels in the first week of ICU stay equal to or greater than 160 mg/dl and the occurrence of dialysis-requiring AKI after adjustment for other confounding variables.

It is estimated that approximately 50% of patients admitted to the ICU have stress hyperglycemia, also referred to as critical illness-related hyperglycemia^3^. Multiple mechanisms are involved in the elevation of blood glucose levels under stress conditions. In addition to the metabolic stress caused by systemic inflammation resulting from sepsis, there is also an increase in the levels of insulin counterregulatory hormones, leading to hyperglycemia^15^. This condition may be exacerbated by pre-existing comorbidities such as diabetes mellitus, obesity, and glucocorticoid use, increasing morbidity and mortality in these patients^15^. In the present study, 63% of patients with sepsis had elevated blood glucose levels, requiring intravenous insulin therapy, although only one-third of the sample had a prior history of diabetes. The characteristics of patients with higher mean blood glucose levels during the first week were similar to those described in other studies involving critically ill patients, in which stress hyperglycemia was more frequent among patients with pre-existing DM, greater disease severity, and use of corticosteroids and vasoactive drugs during ICU stay^1,2,3,4,15^.

The incidence of AKI is high in critically ill patients with sepsis, reaching 40% to 70% of cases^2,3,4,5,6,7^. In this context, AKI is associated with increased mortality, greater need for mechanical ventilation, and longer hospital length of stay^2^. In a multicenter study conducted in 730 ICUs across more than 80 countries^4^, approximately 12% of patients with sepsis developed AKI requiring dialysis – a proportion similar to that found in the present study. The exact mechanism and characteristics of patients who develop AKI in the context of sepsis remain a challenge, as they may involve distinct pathophysiological mechanisms with different prognoses^2^. Furthermore, alterations observed in stress-induced hyperglycemia are also found in patients with sepsis-associated AKI, such as dysregulation of the inflammatory response and increased production of reactive oxygen species^15,16^.

In the present study, patients with mean blood glucose levels ≥160 mg/dL (third tertile) in the first week had a higher risk of dialysis-requiring AKI in the unadjusted analysis; however, this association lost statistical significance after adjustment for intravenous insulin therapy and greater clinical severity (SAPS-3). After adjustment for other confounding variables, no association was found between elevated blood glucose levels and the occurrence of dialysis-requiring AKI. A study involving ward patients admitted for different causes found an association between elevated blood glucose levels on admission and an increased risk of AKI. However, many of these patients had uncontrolled diabetes, with more than 70% having a prior diagnosis of DM^17^. Similarly, a study of 1200 diabetic patients hospitalized for acute myocardial infarction found a threefold increased risk of AKI among those with higher blood glucose levels^12^. Conversely, a clinical trial evaluating stricter glycemic control in critically ill patients with stress hyperglycemia did not demonstrate a protective effect against the occurrence of AKI requiring dialysis^9^. AKI is known to be a multifactorial syndrome involving different pathologies, triggering factors, and patient profiles^2^. Thus, it is believed that the potential deleterious effect of acutely elevated blood glucose levels on tubular cells and on the inflammatory response^10-15,16^ may have been masked by other factors, such as the severity of sepsis itself. Patients with sepsis and greater impairment of other organs may require increased use of vasoactive drugs, mechanical ventilation, and nephrotoxic antibiotics, which may enhance the risk of AKI, regardless of glycemic levels.

This study has limitations that should be considered. First, as a retrospective study based on patient’s data, it is not possible to rule out the risk of information bias due to possible inaccuracies in medical records. However, the ICU studied has a medical residency program and well-defined protocols for data collection and recording, both by the medical team and through interviews with family members. In addition, patients’ continuous-use medications are reviewed by hospital pharmacists, which minimizes the risk of omitting clinical information associated with prior medication use. Due to the limited number of patients with other stages of CKD, it was not possible to assess the effect of the studied exposure on the occurrence of milder stages AKI, which may limit the generalizability of the results. Another limitation of the study was the inability to account for the total dose of insulin or vasoactive drugs administered during ICU stay, which could influence the analysis of the effect of these factors on the occurrence of dialysis-requiring AKI.

Nonetheless, this study evaluated the effect of stress hyperglycemia in a population of patients with well-defined sepsis, according to updated criteria, and considered multiple factors involved in the occurrence of AKI requiring dialysis. This approach may have allowed for a more accurate characterization of the relationship between hyperglycemia and AKI in the context of sepsis.

## CONCLUSION

It is concluded that high mean blood glucose levels in the first week of ICU stay in critically ill patients with sepsis were not associated with the occurrence of AKI requiring dialysis, after adjustment for disease severity and other confounding factors.

## DATA AVAILABILITY

The entire dataset supporting the results of this study is available upon request to the corresponding author, Helbert do Nascimento Lima.
