# Supplementary material for: Association between stress hyperglycemia and acute kidney injury requiring dialysis in critically ill patients with sepsis: a hospital-based cohort study
Source: J Bras Nefrol. 2025 Aug 8;47(4):e20250028. doi: 10.1590/2175-8239-JBN-2025-0028en (PMC12334088; doi:10.1590/2175-8239-JBN-2025-0028en)
Supplement: Translation

# Translation: Associação entre hiperglicemia de estresse e injúria renal aguda dialítica em pacientes críticos com sepse: coorte de base hospitalar

**DOI:** 10.1590/2175-8239-JBN-2025-0028pt

**Authors:** Marina Borges Wageck Horner, Ed Cleso Pereira de Souza, Gustavo Treichel Schelbauer, Lucas de Oliveira Alves, Helbert do Nascimento Lima

**Affiliations:** 1 Universidade da Região de Joinville, Programa de Pós-graduação em Saúde e Meio Ambiente, Joinville, SC, Brasil.; 2 Universidade da Região de Joinville, Faculdade de Medicina, Joinville, SC, Brasil.

**Keywords:** Insuficiência Renal, Hiperglicemia, Sepse, Fatores de Risco

## Abstract

**Introdução:**

A hiperglicemia de estresse em pacientes com sepse não tem sido consistentemente associada a um risco aumentado de injúria renal aguda (IRA).

**Objetivo:**

Avaliar o efeito dos níveis glicêmicos na ocorrência de IRA com necessidade dialítica em pacientes críticos com sepse.

**Métodos:**

Coorte histórica de pacientes com sepse, admitidos na UTI de um hospital privado entre dezembro de 2017 e agosto de 2021. Foram coletadas variáveis clínicas, laboratoriais e de gravidade. Os valores médios da glicemia na primeira semana de UTI (variável de exposição principal) foram estratificados pelos seus tercis. Avaliou-se o efeito da glicemia para a ocorrência de IRA dialítica por meio de regressão logística multivariada.

**Resultados:**

Dos 1317 pacientes avaliados, 86,6% apresentaram situações clínicas como causa de sepse. IRA com necessidade de hemodiálise ocorreu em 12,2% da amostra. Pacientes com médias de glicemia acima do terceiro tercil (≥160 mg/dl), em comparação àqueles com médias de glicemia abaixo dos dois primeiros tercis (<160 mg/dl), tinham maior prevalência de diabetes (69,1% vs. 7,1%; p < 0,001). Pacientes com médias de glicemia ≥160 mg/dl apresentaram uma chance 62% maior de desenvolver IRA com necessidade dialítica, em comparação àqueles com médias de glicemia <160 mg/dl (OR bruto = 1,62; IC 95% 1,16–2,26; p = 0,005). Após ajuste para as demais variáveis, valores médios de glicemia ≥180 mg/dl não aumentaram a chance de ocorrência de IRA (OR = 1,27; IC 95% 0,76–2,12; p = 0,359).

**Conclusão:**

Neste grupo de pacientes, a sepse e níveis médios de glicemia ≥160 mg/dl não foram independentemente associados à ocorrência de IRA dialítica.

## Introdução

Estima-se que 30% das internações em unidades de terapia intensiva (UTI) no Brasil sejam decorrentes de sepse^1^. Dentre as complicações relacionadas à sepse, a hiperglicemia do estresse e a injúria renal aguda (IRA) são frequentemente encontradas nesses pacientes^2,3^. No entanto, o efeito dos valores elevados de glicemia, associado ao estresse, ainda não foi completamente avaliado em relação ao risco de ocorrência de IRA de forma independente.

A sepse é uma importante causa de internação de pacientes adultos em UTIs em todo o mundo^1,2,3,4^. Um estudo conduzido em 730 UTIs, distribuídas em mais de 80 países, demonstrou que a ocorrência de sepse ao longo da internação na UTI foi de 30%, sendo 18% já no momento da admissão^4^. A desregulação do controle glicêmico, denominada hiperglicemia de estresse ou do doente crítico, está presente em 40 a 60% dos pacientes com sepse internados na UTI^3^ e está associada a um maior risco de mortalidade em 30 dias, independentemente da presença de diabetes prévio^5^.

Além da desregulação da glicemia, a sepse está associada a 40 a 70% dos casos de IRA em pacientes admitidos em UTIs^2^, sendo uma importante causa de mortalidade e de aumento do tempo de internação^2,3,4,5,6,7^. Van de Berghe et al.^8^ evidenciaram a importância do tratamento mais restritivo da hiperglicemia (80–110mg/dl), em comparação ao controle glicêmico convencional (180–200mg/dl) em pacientes críticos de uma UTI cirúrgica, na redução das taxas de mortalidade, do tempo de ventilação mecânica e da permanência na UTI, bem como da ocorrência de IRA com necessidade de terapia renal dialítica. O mesmo estudo foi replicado em uma UTI clínica e, apesar da menor ocorrência de IRA nesse cenário, o controle glicêmico mais rigoroso não se mostrou associado a uma menor necessidade de diálise^9^. Estudos em modelos animais têm demonstrado o efeito deletério dos níveis agudamente elevados de glicemia sobre a arquitetura e a homeostase das células tubulares renais, bem como na ativação de mediadores inflamatórios diversos^10^. Estudos envolvendo pacientes críticos internados por insuficiência cardíaca ou infarto têm identificado uma associação entre valores elevados de glicemia, relacionados ao estresse, e a ocorrência de IRA^11,12^. No entanto, essa associação entre hiperglicemia aguda e maior risco de IRA, principalmente no contexto de sepse, não tem sido observada de forma consistente em estudos envolvendo pacientes internados em UTIs^9,10,11,12,13^.

Assim, considerando que a IRA é uma complicação frequente em pacientes internados em UTI, especialmente naqueles cujo motivo de internação foi sepse, o presente estudo teve como objetivo avaliar a associação de hiperglicemia aguda com a ocorrência de IRA com necessidade dialítica em pacientes críticos com critérios bem definidos de sepse.

## Métodos

### Delineamento, Local e Amostragem

Trata-se de uma coorte histórica cujos dados foram obtidos dos prontuários eletrônicos de pacientes internados nas UTIs do Centro Hospitalar Unimed (CHU) de Joinville, SC, no período de dezembro de 2017 a agosto de 2021. O CHU é um hospital geral privado, que atende beneficiários da saúde suplementar Unimed da região. O hospital conta com 152 leitos de enfermaria geral e 19 leitos de UTI geral adulta. Além disso, o CHU oferece programa de residência médica em terapia intensiva, e todos os profissionais médicos que atuam na UTI possuem título de especialização em terapia intensiva. Foram incluídos todos os pacientes com idade igual ou superior a 18 anos, admitidos em uma das UTIs do CHU, com diagnóstico de sepse e/ou choque séptico, com base no critério SEPSE-3^14^. Excluiu-se do estudo pacientes em programa de cuidados paliativos, pacientes com doença renal crônica (história prévia de falência renal crônica já em programa dialítico ou aqueles com creatinina na admissão hospitalar igual ou superior a 3 mg/dl), pacientes com IRA com necessidade dialítica antes da admissão na UTI e aqueles com estadiamento de IRA leve (estágio 1) após a admissão na UTI. Optou-se por excluir pacientes com IRA estágio 1 para focar exclusivamente naqueles com IRA com necessidade dialítica, uma vez que não houve, na amostra estudada, pacientes com IRA estágios 2 ou 3 sem necessidade dialítica. O presente estudo foi aprovado pelo Comitê de Ética da Univille, sob parecer CAAE 51034021.2.0000.5366. O Termo de Consentimento Livre e Esclarecido (TCLE) foi dispensado, considerando-se a natureza retrospectiva e observacional do estudo.

### Variáveis Coletadas

Foram consideradas as variáveis idade, sexo, diagnóstico prévio de diabetes mellitus (DM) informado pelo paciente e motivo de admissão na UTI (causas clínicas ou cirúrgicas). Além disso, considerou-se a necessidade de hemodiálise aguda, o escore de gravidade SAPS 3 (*Simplified Acute Physiology Score 3*) na admissão na UTI, o tempo de ventilação mecânica e o tempo de internação na UTI, bem como o uso de drogas vasopressoras, corticoides e antibióticos nefrotóxicos (vancomicina, anfotericina, aminoglicosídeos e/ou Polimixina B). O uso de insulina também foi considerado, conforme o protocolo institucional de hiperglicemia, no qual dois episódios de hiperglicemia > 180mg/dl são indicativos para o início de insulina endovenosa.

### Principal Variável de Exposição e Desfecho

A glicemia capilar foi verificada por meio do hemoglicoteste (HGT), realizado com medidor de glicose por fita, ambos da empresa G-Tech®. Pacientes em uso de insulina endovenosa tinham HGT verificados a cada hora, enquanto, para os demais, as medições eram realizadas conforme rotina (quatro vezes ao dia). Os valores médios de glicemia na primeira semana na UTI foram considerados como a variável de exposição principal do estudo. A variável de desfecho avaliada foi a ocorrência de IRA estágios 2 ou 3, com ou sem necessidade dialítica de urgência. A definição de IRA foi baseada nos critérios do KDIGO, considerando o aumento da creatinina em relação ao valor basal na admissão na UTI. A indicação de diálise foi definida pelo nefrologista, conforme parâmetros clínicos.

### Análise Estatística

As variáveis categóricas são apresentadas como frequência absoluta e porcentagem. As variáveis numéricas são expressas como média e desvio padrão, ou mediana e variação interquartil. Os valores médios de hiperglicemia na primeira semana de UTI foram estratificados por tercis. Após a verificação de distribuição não normal pelo teste de Kolmogorov-Smirnov, as variáveis quantitativas foram comparadas pelos testes U de Mann-Whitney e Kruskal-Wallis, e as variáveis categóricas, pelo teste qui-quadrado, estratificadas pela exposição principal de interesse e desfecho. Posteriormente, buscou-se comparar, de forma bivariada, o efeito bruto e ajustado de pacientes com valores médios de glicemia na primeira semana acima do terceiro tercil (≥ 160 mg/dl), em relação àqueles com valores entre o primeiro e o segundo tercis (< 160 mg/dl) para ocorrência de IRA com necessidade dialítica. Essa análise foi realizada por meio de regressão logística, considerando as variáveis potencialmente confundidoras. Foram incluídas no modelo multivariado final, por regressão logística, todas as variáveis que modificaram o efeito principal estudado em 5% ou mais, bem como todas aquelas reconhecidamente associadas à exposição principal ou consideradas fatores de risco para o desfecho. Considerou-se o valor de p < 0,05 como estatisticamente significativo. As análises foram realizadas por meio do software estatístico STATA/IC, versão 15.

## RESULTADOS

Dos 3093 pacientes admitidos na UTI no período estudado, 1317 atenderam aos critérios de inclusão e foram considerados na análise, conforme a Figura 1. Desses, 57,6% tinham idade igual ou superior a 60 anos e 53,3% eram homens. A presença de IRA estágio 3 foi encontrada em 12,2% dos pacientes, sendo que todos necessitaram de hemodiálise de urgência. A principal causa de internação na UTI (86,6%) esteve relacionada a condições clínicas. Quando estratificados pela presença ou não de IRA com necessidade de diálise, observou-se que, em comparação aos pacientes sem IRA dialítica, aqueles com IRA dialítica apresentaram maior frequência de idade superior a 44 anos (88,1% vs. 79,7%; p = 0,021) e maior prevalência de homens (66,9% vs. 51,4%; p < 0,001). Além disso, apresentaram valores medianos mais elevados de proteína C reativa (237 mg/dl vs. 183 mg/dl; p < 0,001), creatinina na admissão hospitalar (1,0 mg/dl vs. 0,8 mg/dl; p < 0,001) e na admissão na UTI (1,0 mg/dl vs. 0,7 mg/dl; p < 0,001). Os pacientes com IRA dialítica também necessitaram de maior uso de drogas vasoativas e corticoides, além de apresentarem maior mediana de tempo em ventilação mecânica e de internação hospitalar. As demais características são apresentadas na Tabela 1.

**Figura 1:** Fluxograma da amostra.

**Tabela 1:** Características gerais da amostra, estratificada pela presença ou não de injúria renal aguda (estágio 3)

	Amostra Total n = 1317		Sem IRA n = 1157 (87,8%)		Com IRA n = 160 (12,2%)		Valor *p*
	n° total ou mediana	% ou VIQ	n° total ou mediana	% ou VIQ	n° total ou mediana	% ou VIQ	
Idade, anos							0,021
18–44	254	19,3	235	20,3	19	11,9	
45–59	305	23,2	259	22,4	46	28,7	
≥60	758	57,6	663	57,3	95	59,4	
Sexo, masculino	702	53,3	595	51,4	107	66,9	<0,001
Comorbidades, sim							
Diabetes	424	32,2	363	31,3	61	38,1	0,087
Covid	56	4,2	47	4,0	9	5,6	0,358
Motivo da Admissão							0,136
Clínico	1140	86,6	989	86,4	145	90,6	
Cirúrgico	177	13,4	156	13,6	15	9,4	
Tercil da glicemia média da 1°semana							0,015
70–127	436	33,1	395	34,1	41	25,6	
128–159	433	32,9	384	33,2	49	30,6	
≥160	448	34,0	378	32,6	70	43,7	
PCR admissão UTI, mg/dl	188	66/280	183	62/276	237	83/307	<0,001
Creatinina admissão hospitalar, mg/dl	0,8	0,6/1,2	0,8	0,6/1,1	1,0	0,7/2,1	<0,001
Creatinina admissão UTI, mg/dl	0,8	0,6/1,1	0,7	0,5/1,1	1,0	0,7/2,1	<0,001
IRA (estágio 3), sim	160	12,1					
HD urgência, sim	160	12,1					
SAPS-3	50	42/60	49	42/59	56	49/66	<0,001
VM, dias	6	1/18	4	1/13	20	11/40	<0,001
Dias na UTI	8	3/19	7	3/16	25	14/45	<0,001
Dias hospitalização	16	8/30	14	8/28	33	19/57	<0,001
DVA, sim	586	44,5	484	41,8	102	63,7	<0,001
Corticoide, sim	698	53,0	570	49,3	128	80,0	<0,001
ATB nefrotóxico, sim	193	14,6	118	10,2	75	46,9	<0,001
Insulina EV, sim	827	62,8	703	60,8	124	77,5	<0,001

Abreviações – VIQ: variação interquartil (percentil 25/percentil 75); PCR: proteína C reativa; IRA: injúria renal aguda (estágios 2 ou 3); SAPS-3: *simplified acute physiology score*; VM: ventilação mecânica; DVA: drogas vasoativas; ATB: antibiótico; EV: endovenosa.

A Tabela 2 apresenta as características da amostra, estratificada pelos tercis da média das glicemias na primeira semana na UTI. Houve variação na distribuição etária entre as médias dos tercis de glicemia da primeira semana. Pacientes no terceiro tercil, em comparação ao primeiro tercil, apresentaram maior prevalência de idade acima de 60 anos (70,8 vs. 43,1; p < 0,001), história prévia de diabetes (69,1% vs. 7,1%; p < 0,001) e menor prevalência de internação por causa cirúrgica (8,3% vs. 18,5%; p < 0,001). Além disso, os pacientes no último tercil apresentaram valores mais elevados da mediana da creatinina na admissão hospitalar e na UTI, maior pontuação no escore SAPS-3 de gravidade, maior tempo de internação hospitalar e de permanência na UTI, maior tempo de ventilação mecânica, bem como maior necessidade de uso de drogas vasoativas, corticoides e insulina EV. A presença de IRA com necessidade dialítica também foi maior no grupo com valores médios de glicemia mais elevados na primeira semana, em comparação ao primeiro tercil (15,6% vs. 9,4%; p = 0,015).

**Tabela 2:** Características da amostra estratificada segundo a média dos tercis de glicemia na primeira semana de UTI

	Tercil 1 (70–127 mg/dL) N = 436 (33,1%)		Tercil 2 (128–159 mg/dL) N = 433 (32,9%)		Tercil 3 (≥160 mg/dL) N = 448 (34,0%)		Valor *p*
	Total ou mediana	% ou VIQ	Total ou mediana	% ou VIQ	Total ou mediana	% ou VIQ	
Idade, anos							<0,001
18–44	145	33,3	74	17,1	35	7,8	
45–59	103	23,6	106	24,5	96	21,4	
≥60	188	43,1	253	58,4	317	70,8	
Sexo, masculino	226	51,8	226	52,2	250	55,8	0,424
Comorbidades, sim							
Diabetes	31	7,1	83	19,2	310	69,1	<0,001
Covid-19	14	3,2	22	5,1	20	4,5	0,379
Motivo da Admissão							<0,001
Clínico	353	81,5	375	87,4	406	91,6	
Cirúrgico	80	18,5	54	12,6	37	8,3	
PCR adm UTI, mg/dl	196	68/286	192	70/290	180	59/269	0,127
Creatinina admissão hospitalar, mg/dl	0,7	0,5/1,0	0,8	0,6/1,1	0,9	0,7/1,4	<0,001
Creatinina admissão UTI, mg/dl	0,7	0,5/0,9	0,8	0,6/1,1	0,9	0,6/1,4	<0,001
IRA, sim	41	9,4	49	11,3	70	15,6	0,015
SAPS-3	47	39/55	50	42/60	54	46/64	<0,001
VM, dias	2	1/11	7	1/21	9	3/19	<0,001
Dias UTI	5	3/14	10	4/21	10	5/20	<0,001
Dias hospitalização	13	7/25	18	9/36	18	9/31	<0,001
DVA, sim	152	34,9	196	45,3	238	53,1	<0,001
Corticoide, sim	175	40,1	251	58,0	272	60,7	<0,001
ATB nefrotóxico, sim	61	14,0	66	15,2	66	14,7	0,871
Insulina EV, sim	115	26,4	278	64,2	448	96,9	<0,001

Abreviações – VIQ: variação interquartil (percentil 25/percentil 75); PCR: proteína C reativa; IRA: injúria renal aguda (estágios 2 ou 3); SAPS-3: *simplified acute physiology score*; VM: ventilação mecânica; DVA: drogas vasoativas; ATB: antibiótico; EV: endovenosa.

Quando avaliado o efeito da presença de médias mais elevadas de glicemia na primeira semana (terceiro tercil, ≥160 mg/dl), em comparação aos dois primeiros tercis (<160 mg/dl), quanto à ocorrência de IRA dialítica (Tabela 3), observou-se que pacientes dentro desse cenário apresentaram uma chance 62% maior de desenvolver IRA (OR = 1,62; IC 95% 1,16–2,26; p = 0,005). Após o ajuste de tal efeito sobre a ocorrência de IRA pelas demais variáveis, de forma bivariada, identificou-se que o tempo de ventilação mecânica, o uso de drogas vasoativas, corticoides e drogas nefrotóxicas modificaram esse efeito em 5% ou mais. Quando ajustado para SAPS-3 e necessidade de uso de insulina endovenosa, embora também com mudança do efeito em 5% ou mais, houve perda da associação entre valores médios mais elevados de glicemia na primeira semana acima do terceiro tercil – em comparação aos primeiros dois tercis – e a ocorrência de IRA com necessidade de diálise.

**Tabela 3:** Razão de chance bruta e ajustada (análise bivariada) do efeito das médias de hiperglicemia ≥160mg/dl na ocorrência de IRA dialítica por regressão logística

	OR bruto	IC 95%	Valor p
Efeito da média glicêmica 1° semana no primeiro/segundo tercil vs. terceiro tercil (<160 mg vs. ≥160 mg)			
<160 mg/dl	1,00	Referência	
≥160 mg/dl	1,62	1,16–2,26	0,005
Efeito da média glicêmica 1° semana no primeiro/segundo tercil vs. terceiro tercil (<160 mg vs. ≥160 mg) ajustado por:	OR ajustado		
Idade, anos; por unidade de aumento	1,58	1,12–2,22	0,010
Sexo, homens vs. mulheres	1,59	1,13–2,23	0,007
Diabetes, sim vs. não	1,68	1,19–2,37	0,003
COVID-19, sim vs. não	1,62	1,15–2,26	0,005
PCR admissão UTI, mg/dl; por unidade de aumento	1,64	1,16–2,32	0,005
SAPS-3; por unidade de aumento	1,38	0,95–1,99	0,090
VM, dias; por unidade de aumento	1,51	1,04–2,21	0,032
DVA, sim	1,46	1,04–2,05	0,030
Corticoide, sim	1,43	1,01–2,01	0,042
ATB nefrotóxico, sim	1,72	1,20–2,46	0,003
Insulina EV, sim	1,17	0,80–1,70	0,411

Abreviações PCR: proteína C reativa; SAPS-3: *simplified acute physiology score*; VM: ventilação mecânica; DVA: drogas vasoativas; ATB: antibiótico; EV: endovenosa.

A Tabela 4 apresenta o efeito final da presença de médias glicêmicas acima do terceiro tercil sobre a ocorrência de IRA dialítica. Todas as variáveis previamente analisadas de forma bivariada foram incluídas no modelo, por serem consideradas potenciais confundidoras para a associação principal estudada. Após ajuste para as demais variáveis, a presença de valores médios de glicemia na primeira semana acima do terceiro tercil (≥160mg/dl) não aumentou a chance de ocorrência de IRA com necessidade de diálise (OR = 1,27; IC 95% 0,76–2,12; p = 0,359).

**Table 4:** Análise multivariada do efeito das médias de hiperglicemia ≥160 mg/dl na ocorrência de injúria renal aguda dialítica por regressão logística

	Crude OR	CI 95%	*p-*Value
Effect of mean blood glucose of the 1st week in the first/second tertile vs. third tertile (<160 mg vs. ≥160mg)			
<160 mg/dl	1,00	Reference	
≥160 mg/dl	1 27	0,76–2,12	0,359
Age, Years; per unit of increase	1,00	0,99–1,02	0,618
Sex, men vs. women	1,37	0,86–2,16	0,182
Diabetes, yes vs. no	1,43	0,59–3,52	0,426
COVID-19, yes vs. no	0,98	0,42–2,27	0,963
CRP ICU admission, mg/dl;per unit of increase	1,00	1,00–1,01	0,001
SAPS-3; per unit of increase	1,03	1,01–1,05	0,001
MV, days; per unit of increase	1,02	1,01–1,03	< 0,001
VAD, yes	1,30	0,81–2,08	0,278
Corticosteroid, yes	1,87	1,11–3,16	0,019
Nephrotoxic ATB, yes	4,06	2,47–6,68	< 0,001
IV Insulin yes	1,17	0,65–2,10	0,589

Abbeviations – ICU: intensive care unit; CRP: C-reactive protein; MV: mechanical ventilation; VAD: vasoative drugs.

## Discussão

O presente estudo, baseado em uma coorte histórica de pacientes críticos com critérios bem definidos de sepse, não encontrou associação entre valores médios de glicemia na primeira semana de internação na UTI, iguais ou superiores a 160 mg/dl, e a ocorrência de IRA com necessidade dialítica, após ajuste para outras variáveis de confusão.

Estima-se que cerca de 50% dos pacientes admitidos na UTI apresentem hiperglicemia de estresse, também denominada hiperglicemia da doença crítica^3^. Os mecanismos envolvidos no aumento da glicemia em situações de estresse são diversos. Além do estresse metabólico provocado pela inflamação sistêmica decorrente da sepse, há também elevação dos níveis de hormônios contrarreguladores da insulina, resultando em hiperglicemia^15^. Essa condição pode ser potencializada por comorbidades prévias, como diabetes mellitus, obesidade e uso de glicocorticoides, aumentando a morbimortalidade desses pacientes^15^. No presente estudo, 63% dos pacientes com sepse apresentaram valores elevados de glicemia, necessitando de insulinoterapia endovenosa, embora apenas um terço da amostra tivesse histórico prévio de diabetes. As características dos pacientes com média de glicemia mais elevada na primeira semana foram semelhantes às descritas em outros estudos envolvendo pacientes críticos, nos quais a hiperglicemia de estresse foi mais frequente entre aqueles com DM prévio, maior gravidade e uso de corticoides e de drogas vasoativas durante a internação na UTI^1,2,3,4,15^.

A presença de IRA é altamente incidente em pacientes críticos com sepse, atingindo de 40% a 70% dos casos^2,3,4,5,6,7^. Nesse contexto, a IRA está associada ao aumento da taxa de mortalidade, da necessidade de ventilação mecânica e do tempo de internação^2^. Em um estudo multicêntrico, realizado em 730 UTIs de mais de 80 países^4^, aproximadamente 12% dos pacientes com sepse desenvolveram IRA com necessidade dialítica – proporção semelhante à encontrada no presente estudo. O mecanismo exato e as características dos pacientes que evoluem com IRA no contexto de sepse ainda representam um desafio, uma vez que podem envolver mecanismos fisiopatológicos distintos, com diferentes prognósticos^2^. Além disso, alterações encontradas na hiperglicemia induzida pelo estresse também são observadas em pacientes com IRA associada à sepse, como desregulação da resposta inflamatória e aumento da produção de espécies reativas de oxigênio^15,16^.

No presente estudo, pacientes com média de glicemia na primeira semana ≥160 mg/dL (terceiro tercil) apresentaram maior risco de IRA com necessidade de diálise na análise bruta; porém, essa associação perdeu significância estatística após ajuste pelo uso de insulinoterapia endovenosa e maior gravidade do quadro clínico (SAPS-3). Após ajuste para as demais variáveis de confusão, não foi encontrada associação entre níveis elevados de glicemia e a ocorrência de IRA dialítica. Um estudo envolvendo pacientes internados em enfermaria por diferentes causas encontrou associação entre níveis elevados de glicemia na admissão e maior risco de IRA. No entanto, grande parte desses pacientes apresentava diabetes não controlado, sendo que mais de 70% tinham diagnóstico prévio de DM^17^. De forma semelhante, um estudo com 1200 pacientes diabéticos internados por infarto agudo do miocárdio encontrou um risco três vezes maior para IRA entre aqueles com níveis mais elevados de glicemia^12^. Por outro lado, um ensaio clínico que avaliou um controle glicêmico mais rigoroso em pacientes críticos com hiperglicemia de estresse não demonstrou efeito protetor para a ocorrência de IRA com necessidade de diálise^9^. Sabe-se que a IRA é uma síndrome multifatorial, envolvendo diferentes patologias, fatores desencadeantes e perfis de pacientes^2^. Dessa forma, acredita-se que o possível efeito deletério dos níveis agudamente elevados de glicemia sobre as células tubulares e sobre a resposta inflamatória^10,11,12,13,14,15,16^ pode ter sido mascarado por outros fatores, como a própria gravidade da sepse. Pacientes com sepse e maior comprometimento de outros órgãos podem apresentar maior necessidade de drogas vasoativas, ventilação mecânica e antibióticos nefrotóxicos, o que potencializa o risco de IRA, independentemente dos níveis glicêmicos.

Este estudo apresenta limitações que devem ser consideradas. Primeiramente, por se tratar de um estudo retrospectivo, baseado em dados de prontuário, não é possível afastar o risco de viés de informação decorrente de possíveis falhas no registro médico. No entanto, a UTI estudada conta com programa de residência médica e com protocolos bem definidos para coleta e registro de dados, tanto por parte da equipe médica quanto por meio de entrevistas com familiares. Além disso, as medicações de uso contínuo dos pacientes são revisadas por farmacêuticos hospitalares, o que minimiza o risco de omissão de informações clínicas associadas ao uso prévio de medicamentos. Devido ao número limitado de pacientes com outros estágios de DRC, não foi possível avaliar o efeito da exposição estudada na ocorrência de IRA em estágios mais leves, o que pode limitar a generalização dos resultados. Outra limitação do estudo foi a impossibilidade de considerar a dose total de insulina ou de drogas vasoativas administradas durante a internação na UTI, o que poderia influenciar a análise do efeito desses fatores sobre a ocorrência de IRA com necessidade dialítica.

Ainda assim, trata-se de um estudo que avaliou o efeito da hiperglicemia de estresse em uma população bem definida de pacientes com sepse, segundo critérios atualizados, e que considerou múltiplos fatores envolvidos na ocorrência de IRA com necessidade dialítica. Essa abordagem pode ter permitido uma caracterização mais precisa da relação entre hiperglicemia e IRA no contexto da sepse.

## Conclusão

Conclui-se que valores médios elevados de glicemia na primeira semana de internação na UTI, em pacientes críticos com sepse, não foram associados à ocorrência de IRA com necessidade dialítica, após ajuste para gravidade da doença e outros fatores de confusão.

## DISPONIBILIDADE DE DADOS

Todo o conjunto de dados que dá suporte aos resultados deste estudo está disponível mediante solicitação ao autor correspondente Helbert do Nascimento Lima.
